# Metabolic characteristics of the various incision margins for breast cancer conservation surgery

**DOI:** 10.3389/fonc.2022.959454

**Published:** 2023-01-04

**Authors:** Fang Wang, Zongze Gu, Xunan Zhao, Zhuo Chen, Zhe Zhang, Shihao Sun, Mingli Han

**Affiliations:** Department of Breast Surgery, The First Affiliated Hospital of Zhengzhou University, Zhengzhou, China

**Keywords:** breast cancer, breast-conserving surgery, surgical margin, metabolomics, potential biomarker

## Abstract

**Background:**

Breast cancer (BC) has recently become the most prevalent malignancy in women. There are many alternative treatments for BC, and for aesthetic and postoperative quality of life concerns, breast-conserving surgery and corresponding adjuvant therapy have become the predominant treatment for early invasive BC. Currently, the main method used to assess the margins for breast-conserving surgery is intraoperative pathological diagnosis. However, the designation of surgical margins is controversial, and metabolomics may be a novel approach to evaluate surgical margins.

**Methods:**

We collected specimens from 10 breast cancer patients and samples from its surrounding tissues and divided them into cancerous tissue and 1 mm, 2 mm, 3 mm, 5 mm and 10 mm cutting edge tissues, with a total of 60 samples. The samples were analyzed by mass spectrometry on an ultra-performance liquid chromatography-quadrupole/Orbitrap high resolution platform. The data were then statistically analyzed to detect metabolic changes in the different cutting edges and to identify possible surgical cutting edges with statistically significant findings. Abnormal metabolic pathways were identified by Kyoto Encyclopedia of Genes and Genomes (KEGG), which elucidated potential markers.

**Results:**

Statistical analysis indicated that there were substantial differences between the 1 mm margin tissue and the cancer tissue, while there were no statistically significant differences between the 1 mm tissue and tissues from the other margins. The levels of 6 metabolites in the 1 mm tissue were significantly different from those in the cancer tissue and were not significantly different from those in the 2 mm tissue. The six metabolites were pyruvate, N-acetyl-L-aspartate, glutamic acid, γ-aminobutyric acid, fumaric acid, and citric acid. Metabolic pathways such as amino acid metabolism and amino t-RNA synthesis in the margin tissue were significantly distinct from those in cancer tissues based on KEGG analysis.

**Conclusion:**

There was a significant difference between the 1 mm margin tissue and the cancerous tissue. Based on metabolomic analysis, the 1 mm negative margin is sufficient for surgery, and the six metabolites that we identified as abnormal, including pyruvic acid, N-acetyl-L-aspartic acid, glutamic acid, gamma-aminobutyric acid, fumaric acid and citric acid, may serve as biomarkers for a negative margin and help surgeons select an appropriate surgical margin.

## Introduction

Among the malignancies to occur in women in recent years, breast cancer is the most common malignancy in terms of incidence (1), which has exhibited a slow increase of approximately 0.5% per year since 2000; this increase is related to the current decline in fertility and the increasing prevalence of overweightness in society (2), and breast cancer is the leading cause of death from malignancies in women aged 20 to 59 years (1). There are many treatment options available for breast cancer, with surgery, radiotherapy, chemotherapy, endocrine therapy and immunosuppressive agents all offering a good chance of survival. Breast-conserving surgery and the corresponding adjuvant treatment are now the accepted treatment for early invasive breast cancer, providing patients with better quality of life and cosmetic results and more psychological benefits than traditional radical surgery. It has been demonstrated through various trials that breast-conserving surgery does have acceptable morbidity and mortality rates (3, 4). There are many factors that influence the recurrence and prognosis of breast cancer, such as pathological classification, tumor size, presence of distant metastases and depth of infiltration; additionally, regarding breast-conserving surgery, the impact of the surgical margins should not be underestimated (5, 6).

The designation of surgical margins for breast-conserving surgery has long been controversial, with a wider margin affecting the patient’s postoperative aesthetics and a narrower margin increasing the risk of reoperation and local recurrence. Some studies have shown that most surgeons currently prefer a 2 mm margin (7). However, guidelines suggest that with good preoperative diagnostic and ancillary techniques, excessive excision of healthy tissue is of no better benefit, leading to the “no tumor ink” guideline (8). Currently, the main method used to diagnose negative margins is intraoperative freezing as judged by the pathologist, but this technique has limitations and can also increase the duration of the procedure, with a considerable physical and financial impact on the patient. In recent years, new techniques have also emerged, such as microcomputed tomography for intraoperative assessment (9). However, this technique has not been widely used in clinical practice. We applied metabolomics to analyze and assess the surgical margins and identify possible surgical margins and potential markers. However, metabolomics has certain limitations that are not associated with traditional intraoperative rapid frozen pathology and other detection methods. The preprocessing of tissue specimens and the processing of data after mass spectrometry analysis take a longer time and do not provide timely feedback to clinicians; additionally, the technology is more costly, which increases the financial pressure on patients.

A distinctive feature of cancer is metabolic reprogramming, whereby cancer tissues exhibit altered metabolic pathways to adapt to their environment and meet their own growth requirements; for example, cancer tissues can preferentially undergo anaerobic glycolysis under aerobic conditions (10), which is a phenomenon known as the “Warburg effect”. Metabolomics has been extensively used to study breast cancer, offering novel approaches to its diagnosis, treatment and prognosis. Triple-negative breast cancer, which is a substantial challenge, is characterized by a high recurrence rate, few treatment options and a poor prognosis (11, 12). Jiang et al. (13, 14) have provided new possibilities for triple-negative breast cancer through metabolomics studies. Research has shown that triple-negative breast cancer can be classified into three types, namely, the lipogenic subtype, glycolytic subtype and mixed subtype, based on the main abnormal metabolic pathways (13), with various subtypes exhibiting different sensitivities to different treatments. This typing can provide new therapeutic tools; studies investigating these approaches are unlike the numerous studies aiming to identify a more precise method for pathological subtyping (15, 16). Metabolomics has been understudied in the context of breast-conserving surgery; thus, we used metabolomics techniques to analyze different incision margins to provide the possibility for a negative incision margin for breast-conserving surgery.

In this study, we used ultrahigh-performance liquid chromatography-quadrupole/Orbitrap high-resolution mass spectrometry (UHPLC-Q-Orbitrap HRMS) to metabolically analyze 60 specimens that were collected and then statistically analyzed the data to identify tissue at cut edges with significant differences from the tumor tissue and to identify potential markers that might be present.

## Materials and methods

### Sample collection

After obtaining informed consent from patients, we collected cancer tissue specimens from 10 breast cancer patients at the First Affiliated Hospital of Zhengzhou University and samples from the surrounding tissue in 2021 and maintained them in a -80°C refrigerator until they were machine-processed and examined. We separated each specimen into six groups of cancer tissues, tissues with a 1 mm cut edge, tissues with a 2 mm cut edge, tissues with a 3 mm cut edge, tissues with a 5 mm cut edge and tissues with a 1 cm cut edge, for a total of 60 samples for UHPLC‒MS/MS processing.

### Sample preparation

First, we weighed each specimen, added 100 µl of pure methanol solution to 10 mg of tissue, added small steel beads and placed the samples into a grinder (SCIENTZ-48 high throughput tissue grinder) for 30 minutes. After removal, each sample was placed in a centrifuge (Centrifuge CF16RN HITACHI, Tokyo, Japan) and centrifuged at 3000 rpm for 30 minutes at 4°C. After extraction of the supernatant, the specimens were concentrated in a concentrator, removed and added to 300 µl of pure methanol solution and placed in a redissolution machine (Mutil-Tube Vortexer) at 2500 rpm for 3 minutes. Then, we placed the samples in a centrifuge at 3000 rpm for 30 minutes at 4°C. One hundred microliters of supernatant was extracted from each sample and transferred to an autosampler vial for in-machine UHPLC‒MS/MS processing. The reproducibility and reliability of the UHPLC‒MS/MS system was assessed by means of quality control (QC) samples.

### UHPLC-Q-Orbitrap HRMS analysis

We used an ultra-performance liquid chromatography system (Thermo Fisher Scientific Dionex, Waltham, Massachusetts, USA) and an Acquity UHPLC BEH C18 column (2.1 mm × 100 mm, 1.7 µm, Waters, USA) to achieve chromatographic separation and gradient elution. We used acetonitrile as mobile phase A and water containing 0.1% formic acid as mobile phase B at a flow rate of 0.2 mL/min. The elution gradient was employed as follows: 0–0.5 min, 5% A, 0.5–2 min, 5–40% A; 3–8 min, 40–60% A; 9–11 min, 80–90% A, 12–13 min, 90–100% A, 14–15 min, 100% A.

- We performed MS separations in full scan mode using a Q-Orbitrap mass spectrometer equipped with thermoelectric spray ionization (HESI) (Thermo Scientific, San Jose, USA). We used a mass spectrometer in full scan mode to obtain positive and negative mode mass spectra. Substances in the mass range of 80 to 1200 m/z could be scanned by the instrument. The speed of the auxiliary gas was set to 10 arb, and the temperature was set to 300°C. The capillary temperature was set to 320°C. The spray voltages in positive and negative mode were set to 3.5 kV and 2.8 kV, respectively.

### Data processing

We used Compound Discoverer 3.1 software (version 3.0, Thermo Scientific) to extract metabolites from the raw data file to generate a comprehensive peak table containing retention times (RT), molecular weights and peak areas. The data were then visualized using Xcalibur™ (Version 3.0, Thermo Fisher Scientific) software and compared to the Human Metabolomics Database (HMDB, http://hmdb.ca/) to identify metabolites from different sources. The metabolites screened by the HMDB were then subjected to enrichment analysis using the KEGG database to identify pathways with *p <*0.05 and a false discovery rate (FDR) <0.05.

### Statistical analysis

Each sample corresponding to a metabolite contains m/z values, ion peak areas and RT. We used SIMCA software (Version 14.0 Umetrics, Umea, Sweden) to perform principal component analysis (PCA) and orthogonal partial least squares (OPLS-DA) and to obtain projected variable importance (VIP) values. Fold change and t test (*p* value < 0.05, FDR < 0.05) results were obtained by MetaboAnalyst (https://www.MetaboAnalyst.ca/). Receiver operating characteristic (ROC) curves and area under the curve (AUC) were used to assess the sensitivity and accuracy of analyses performed using the metrics.

## Results

### Clinical characteristics of the breast cancer patients

We collected tissue samples from 10 clinical breast cancer patients and the adjacent tissue and divided the 60 samples into 6 groups, including tumor tissue and tissues located 1 mm, 2 mm, 3 mm, 5 mm and 10 mm from the tumor margin, for analysis by UHPLC‒MS/MS. The clinical characteristics of the 10 patients are shown in **Supplementary Table 1**.

### Metabolomic analysis

**Figure 1:**
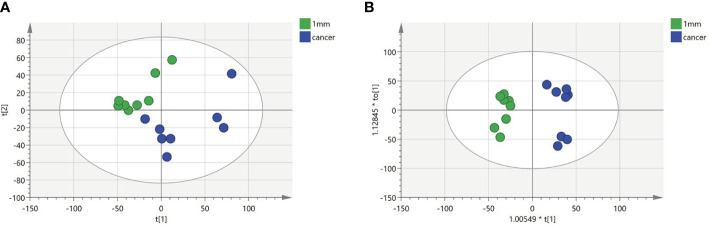
Multivariate statistical analysis of two groups. Principal component analysis (PCA) plot comparing between 1 mm cut edge tissue and cancer tissue in **(A)** negative ion mode; orthogonal partial least squares discriminant analysis (OPLS-DA) score plots comparing between 1 mm cut edge tissue and cancer tissue in **(B)** negative ion mode.

**Figure 2:**
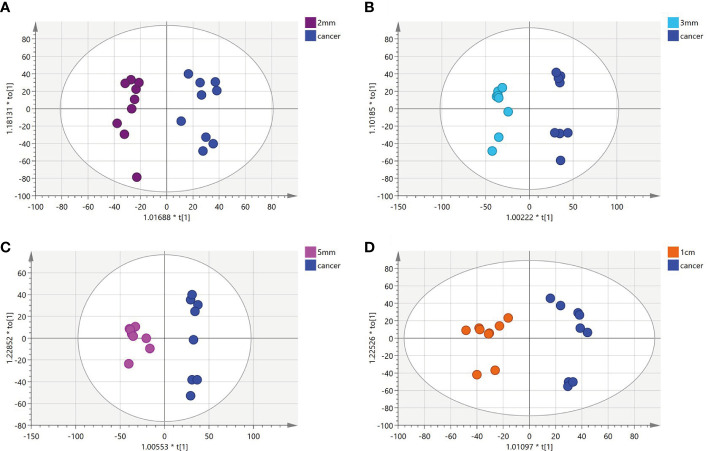
**(A-D)** Orthogonal partial least squares discriminant analysis (OPLS-DA) score plots comparing between tissue located at other surgical margins and cancer tissue.

**Figure 3:**
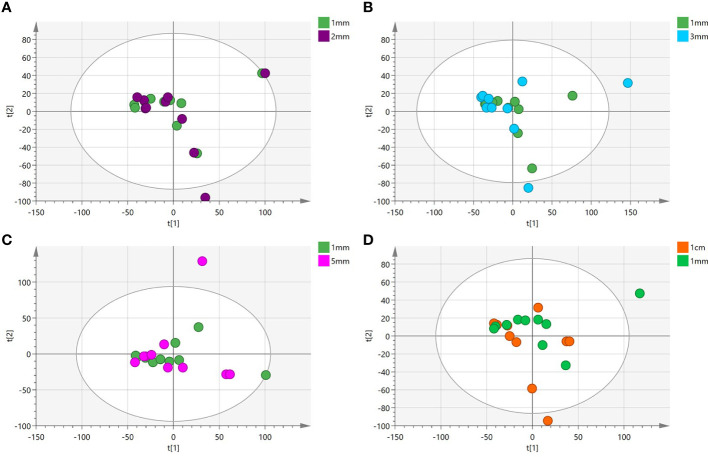
**(A-D)** Principal component analysis(PCA) plot comparing between tissue located at the 1mm surgical margin and other surgical margins.

**Figure 4:**
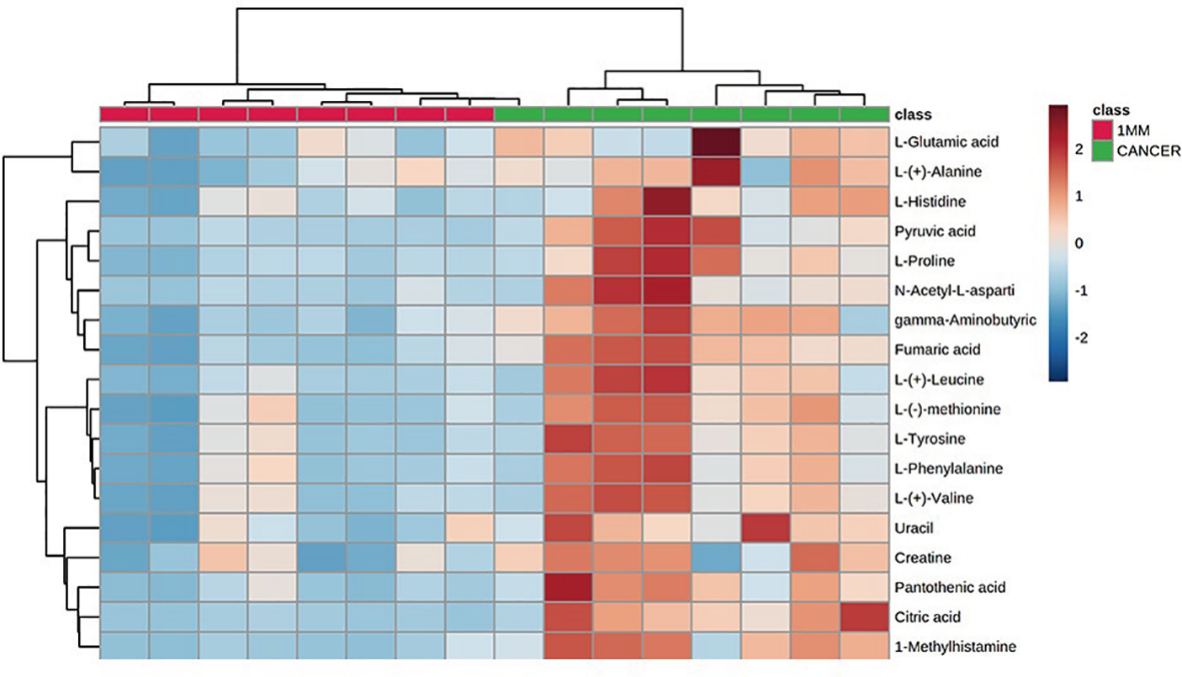
Heatmap indicating the relative levels of statistically significant differential metabolites in 1-mm cut edge tissue and cancerous tissue.

**Figure 5:**
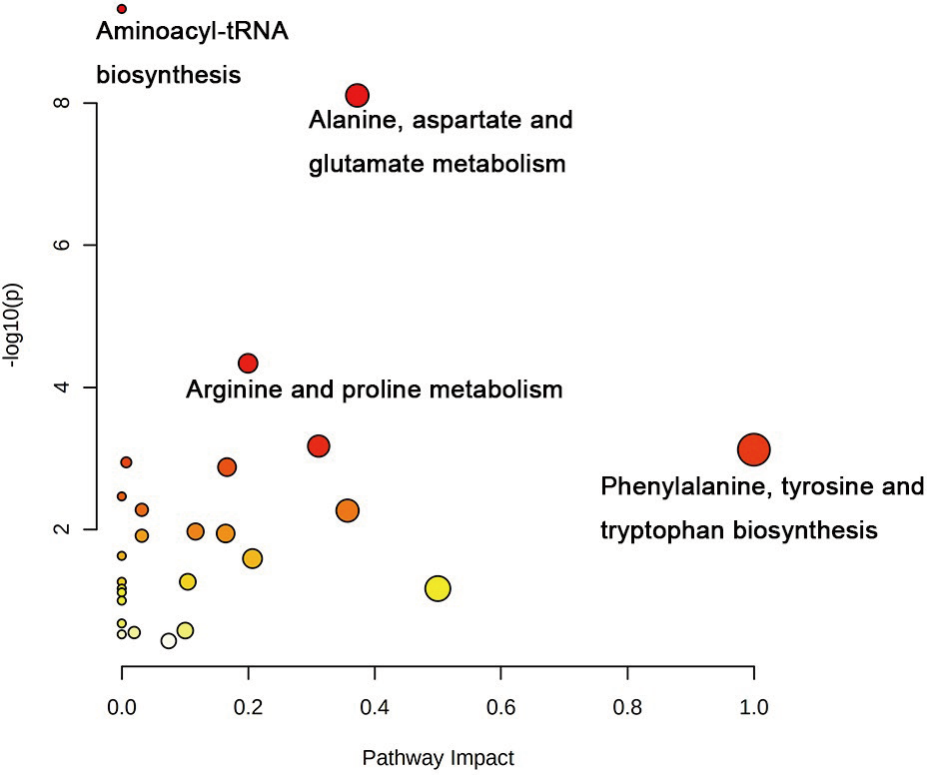
Correlation network analysis of metabolites identified in untargeted metabolomics. Correlation analysis of 18 differential metabolites with statistical differences between 1 mm surgical margin and cancerous tissue.

To identify a possible negative cut edge, we performed UHPLC‒MS/MS analysis on the tumor tissue and tissues from different cut edges, analyzed and processed the data, and imported the analyzed data into SIMCA version 14.0 for statistical analysis. First, we performed PCA on the data obtained from the 1 mm cut edge and data obtained from cancer tissue in negative ion mode (**Figure 1A** **)**. Immediately afterward, we performed OPLS-DA (**Figure 1B** **)** with an R2Y of 0.96 and a Q2 of 0.801. Significant detachment was found between the cancerous tissue and the 1 mm incision margin tissue, with the same result observed in the positive ion mode; these data are shown in **Supplementary Table 2** and **Supplementary Figure S1**. There was also apparent separation between other margin tissues and cancerous tissues (**Figure 2** **)**. The variation among the different cut edge tissues was analyzed by PCA **(** **Figure 3** **)**. The data showed no statistically significant difference between the tissue at the 1 mm margin and the tissue at the other margins, so we designated the margin closest to the cancerous tissue with a statistically significant difference as the possible negative margin, which was the 1 mm margin. We identified over 50 endogenous differentially expressed metabolites from a set of metabolites, with *P <*0.05, VIP>1.0 and fold change>1.5 as the cutoff. We screened the metabolites with statistically significant differences in levels between the 1 mm cut margin tissue and cancer tissue by KEGG, and the results of the statistically significant aberrant metabolic pathways found to be enriched are shown in **Table 1**; the results indicated that 18 differential metabolites were enriched in statistically significant aberrant metabolic pathways. The statistically significant abnormal metabolic pathways included those for amino acids such as glutamate, alanine, aspartate, histidine, arginine, proline, glutamine, tyrosine and phenylalanine, abnormal synthesis of aminyl-tRNA, and abnormal pantothenic acid biosynthesis. We generated a heatmap of the levels of metabolites capable of being enriched in statistically significant aberrant metabolic pathways by MetaboAnalyst to demonstrate the differences in metabolite levels between 1 mm cut edge tissue and cancerous tissue (**Figure 4** **)**. The topological analysis of the aberrant metabolic pathways indicated that amino acid metabolism was the main deviant metabolic pathway in cancerous tissues (**Figure 5** **)**.

**Table 1:** Statistically significant metabolic pathways that differed between 1 mm cut margin tissue and cancer tissue identified by KEGG analysis.

Metabolite Set	Total	Hits	Expect	*P* Value	FDR
Alanine, aspartate and glutamate metabolism	28	7	1.34	2.31E-4	0.0127
Aminoacyl-tRNA biosynthesis	48	9	2.3	2.31E-4	0.0127
Pantothenate and CoA biosynthesis	19	4	0.911	0.0109	0.269
Phenylalanine, tyrosine and tryptophan biosynthesis	4	2	0.192	0.0128	0.269
D-Glutamine and D-glutamate metabolism	6	2	0.288	0.03	0.451
Arginine and proline metabolism	38	5	1.82	0.0322	0.451
Histidine metabolism	16	3	9.767	0.0378	0.451

### Identification of potential markers

**Figure 6:**
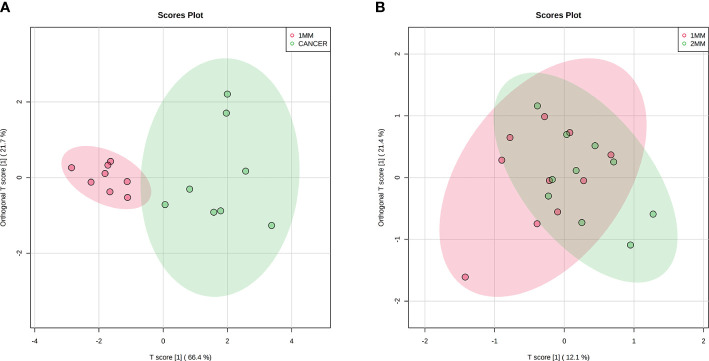
Orthogonal partial least squares discriminant analysis (OPLS-DA) score plots of 6 potential biomarkers in tissue located at 1 mm surgical margin and cancerous tissue **(A)**, showing a clear separation. Orthogonal partial least squares discriminant analysis (OPLS-DA) score plots of 6 potential biomarkers in tissue located at the 1 mm surgical margin and 2 mm surgical margin **(B)**, with no significant difference.

**Figure 7:**
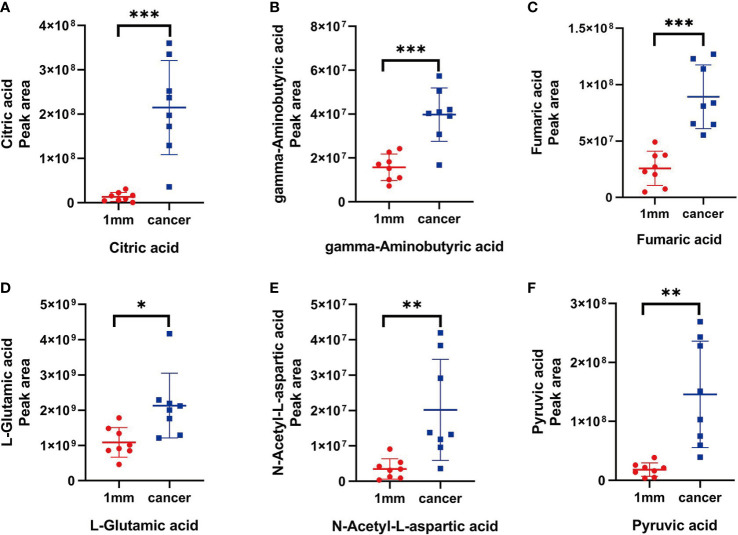
**(A-F)** The peak areas of citric acid, gamma-aminobutyric acid, fumaric acid, L-glutamic acid, N-acetyl-L-aspartic acid and pyruvic acid in tissue located at the 1 mm surgical margin and tumor tissue, with significant differences (P < 0.05)."*" stands for the p < 0.05,"**" stands for the p < 0.01, "***" stands for the p < 0.001.

**Figure 8:**
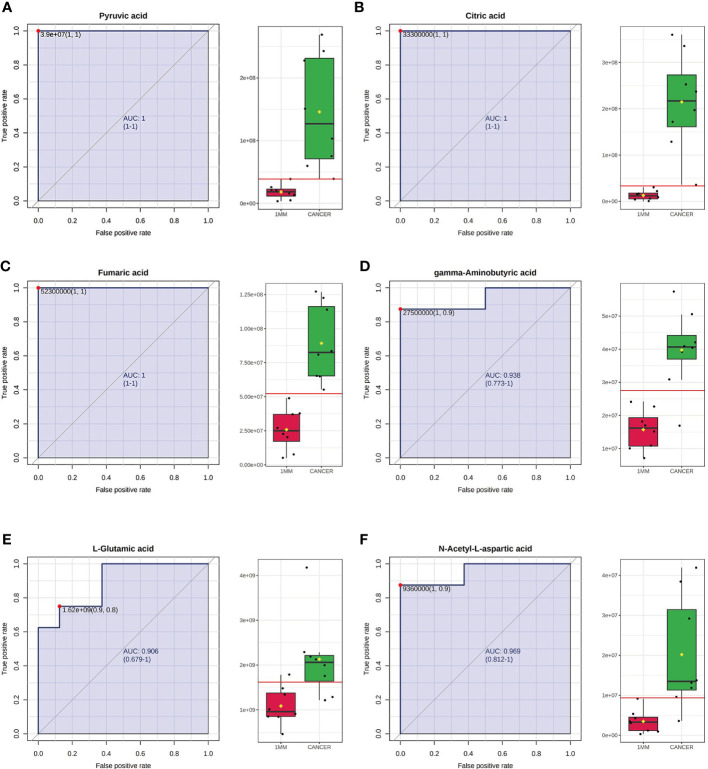
Receiver operating characteristic (ROC) curves using the levels of pyruvic acid **(A)**, citric acid **(B)**, fumaric acid **(C)**, gamma-aminobutyric acid **(D)**, L-glutamic acid **(E)** and N-acetyl-L-aspartic acid **(F)**. The AUCs obtained using the levels of pyruvic acid, citric acid, fumaric acid, gamma-aminobutyric acid, L-glutamic acid and N-acetyl-L-aspartic acid were 1 (95% CI=1-1), 1 (95% CI=1-1), 1 (95% CI=1-1), 0.938 (95% CI=0.773-1), 0.906 (95% CI=0.679-1) and 0.969 (95% CI=0.812-1), respectively. The box plots show the median, quartiles, and whole range of peak areas of the levels of these metabolites.

We selected six metabolites from a large number of metabolites with statistically significant differences in levels (*p* value <0.05, VIP >1.0 and included in all statistically significant differences between margin tissue and cancer tissue). The OPLS-DA of N-acetyl-aspartate, alanine, glutamic acid, aminobutyric acid, citric acid and fumaric acid values were found to be separated between the 1-mm cut edge tissue and the cancer tissue (**Figure 6A** **)**. We also performed OPLS-DA of these six metabolites using the 1-mm cut edge tissue and the 2-mm cut edge tissue and found no significant difference between them **(** **Figure 6B** **)**. The information for these six metabolites is shown in **Table 2**. We then analyzed the differences in the levels of each of these six metabolites between the cancerous tissue and the 1-mm cut edge tissue (**Figure 7** **)**. As expected, each metabolite had significantly different levels between the cancerous tissue and the 1-mm cut edge tissue. This finding was consistent with our expectations, suggesting that these differential metabolites may be potential markers of negative cut margins. We tested the ability of these six metabolites to distinguish between cancer and negative margins by plotting ROC curves using the levels of five metabolites, which resulted in an AUC > 0.9 and one metabolite with an AUC > 0.8 (**Figure 8** **)**, demonstrating the good sensitivity and specificity of these indicators.

**Table 2:** Statistical analysis of potential metabolic biomarkers.

No.	Metabolities	Lon mode	RT(min)	Molecular	VIP	*P* Value
**1**	Pyruvic acid	N	1.407	88.01493	1.80	0.001385
**2**	N-Acetyl-L-aspartic acid	N	1.432	175.0479	1.57	0.005883
**3**	L-Glutamic acid	N	0.938	147.052	1.80	0.000828
**4**	gamma-Aminobutyric acid	N	0.963	103.0637	1.85	0.000196
**5**	Fumaric acid	N	1.630	116.0098	1.94	0.0000652
**6**	Citric acid	N	1.433	192.0262	1.97	0.000104

RT, retention time; VIP, variable importance in projection.

## Discussion

Breast cancer is already the most prevalent cancer in women in today’s society (1); although there are many treatments available, breast-conserving surgery is one of the accepted treatments for early invasive breast cancer, with most surgeons preferring a 2-mm margin (7). The Society of Surgical Oncology (SSO) and the American Society of Radiation Oncology (ASTRO)-American Society of Clinical Oncology (ASCO) consensus guidelines state that the principle of “no tumor ink” is recommended for breast-conserving surgical margins in stage I and II invasive breast cancer, which can also achieve a reduction in local recurrence rates (8). The main method used to diagnose cut margins is currently intraoperative rapid cytopathology; however, this method has limitations and is less sensitive than conventional pathology using paraffin blocks (17). Other techniques are also used in the diagnosis of cut edges, such as mammography, intraoperative breast ultrasound, the adjunctive use of magnetic resonance imaging (MRI) techniques and, in recent years, optical techniques and isotope methods. Macroscopic margin assessment is also a diagnostic method used to evaluate cutting edges (18–20); however, these methods have limitations and have developed slowly.

Metabolomics is a field that has experienced rapid growth in recent years and has a promising future as a complement to genomics, proteomics and other “downstream” omics approaches. Metabolomics has great potential for use in relatively noninvasive liquid tests that can be used in the diagnosis and prognosis of cancer (21). In this study, we investigated the metabolism of different cut edge tissues and cancer tissues using 10 breast cancer samples by UHPLC‒MS/MS and found statistically significant differences between 1 mm cut edge tissues and cancer tissues and no statistically significant differences between 1 mm cut edge tissues and other tissues from the remaining cut edges. We also identified six metabolites involved in abnormal metabolism (P<0.05, VIP>1.0, AUC>0.8) that could be potential markers for identifying negative incision margins. However, metabolomics has certain limitations that are not associated with traditional intraoperative rapid frozen pathology and other detection methods. The preprocessing of tissue specimens and the processing of data after mass spectrometry analysis take a longer time and do not provide timely feedback to clinicians; additionally, the technology is more costly, which increases the financial pressure on patients.

In the present study, amino acid metabolism was significantly abnormal in the tumor tissue, and many diseases are known to be associated with abnormal amino acid metabolism (22, 23). We found significantly higher concentrations of glutamate, which plays an important physiological role in the body as a nonessential amino acid and excitatory neurotransmitter, in the cancer tissues in this study. It has been shown that glutamate levels are significantly elevated in cancer tissues (24). Glutamate is produced from glutamine by the action of glutaminase, an enzyme found in the internal mitochondrial membrane, and it has been shown that glutaminase activity is increased by its overexpression in cancer tissues (25, 26), leading to increased levels of glutamate in cancer tissues, which is consistent with our findings.

Aspartate metabolism was found to be significantly active in the cancer tissues in this study, and concentrations were significantly increased in the cancer tissues. It has been suggested that aspartate, asparagine and asparagine synthase may be potential markers for surgical cutting edges in oral squamous cell carcinoma (27), and studies have found significantly higher concentrations of aspartate and significantly increased activity of asparagine synthase in cancer tissue; the exact mechanism underlying this phenomenon is unclear, as indicated by our experimental results.

In addition to abnormal amino acid metabolism, abnormal aminyl-tRNA biosynthesis in cancer tissues was a distinctive feature of the results in this study. Aminyl-tRNA biosynthesis has been found to be significantly elevated in metabolomic studies of gastric cancer (28), in which metabolomic and bioinformatics analysis of gastric and paracancerous tissues revealed that aminyl-tRNA biosynthesis exhibited abnormally increased activation in gastric cancer tissues, the expression level of phenylalanine-tRNA synthetase was associated with poor survival, and the expression level of threonine-tRNA synthetase was associated with tumor grade. In addition, amyl-tRNA synthetase and its interacting proteins play an important role in tumorigenesis (29, 30), suggesting that the amyl-tRNA biosynthetic pathway offers a new possibility for limiting tumor growth in the future.

In conclusion, by using UHPLC‒MS/MS to investigate the metabolism of different marginal tissues and cancerous tissues from 10 breast cancer specimens, we identified the 1-mm margin as a possible margin for breast-conserving surgery. The results of this study are limited in that we collected specimens from only 10 breast cancer patients, which is a small sample size. This study is a preliminary study that performed an initial examination of the surgical margins of breast-conserving surgery from a metabolomics perspective to provide possibilities for clinicians. We have also identified six potential markers, but the value of these markers has yet to be further validated, and we will recruit more clinical patients in the future for further research and to validate the results. Therefore, the method is still experimental and has certain limitations. The pretreatment of tissues and data processing after mass spectrometry analysis require a longer time, and the expensive cost is also a nonnegligible problem, so the method may take a long time to enter clinical practice.

## Data availability statement

The raw data supporting the conclusions of this article will be made available by the authors, without undue reservation.

## Ethics statement

The studies involving human participants were reviewed and approved by Ethics Committee of Scientific Research Project of The First Affiliated Hospital of Zhengzhou Unive. The patients/participants provided their written informed consent to participate in this study.

## Author contributions

MH and FW designed the study. ZG and SS carried out the experiments. ZG and XZ undertook the data analysis and wrote the manuscript. ZC and ZZ assisted in Data curation and editing the manuscript. All authors contributed to the article and approved the submitted version.
