# Supplementary material for: Metabolic characteristics of the various incision margins for breast cancer conservation surgery
Source: Front Oncol. 2023 Jan 4;12:959454. doi: 10.3389/fonc.2022.959454 (PMC9846322; doi:10.3389/fonc.2022.959454)

frontiers

Supplementary Material

Supplementary Table S1. The baseline characteristics of 10 patients

		Number	Percent (%)
Age	<50	6	60
	≥50	4	40
Tumor size	<2cm	4	40
	≥2cm	6	60
Lymph node metastasis	Yes	5	50
	No	5	50
pTNM stage	StageI	2	20
	StageII	5	50
	StageIII	3	30
Estrogen receptor expression status	Positive	5	50
	Negative	5	50
Progesterone receptor expression status	Positive	4	40
	Negative	6	60
Human epidermal growth factor receptor-2 expression status	Positive	3	30
	Negative	7	70

Supplementary Table S2. Statistically significant differential metabolites between 1mm cut edge tissue and cancer tissue in positive mode

**No.**	**Metabolities**	**Lon mode**	**RT(min)**	**Molecular**	**VIP**	***P*-Value**
1	L-Isoleucine	P	1.643	131.0943	1.58795	0.007371
2	L-Tyrosine	P	1.518	181.0735	1.5784	0.006691
3	L-Glutamic acid	P	0.94	147.0529	1.5933	0.007623
4	L-Methionine	P	1.446	149.0509	1.55549	0.00936
5	Valine	P	1.442	117.079	1.56588	0.007953
6	L-Histidine	P	0.835	155.0693	1.75424	0.001712
7	Uracil	P	1.039	112.0275	1.66839	0.002513
8	Sphingosine	P	7.548	299.2818	1.65345	0.007243
9	Pantothenic acid	P	3.381	219.1105	1.81361	0.00072
10	Phosphoethanolamine	P	0.933	141.019	1.83679	0.000516
11	Sphinganine	P	7.706	301.2975	1.57676	0.009767

Supplementary Figure S1. Principal component analysis (PCA) plot between 1mm cut edge tissue and cancer tissue in (A) positive ion mode, Orthogonal partial least squares discriminant analysis (OPLS-DA) score plots between 1 mm cut edge tissue and cancer tissue in (B) positive ion mode.

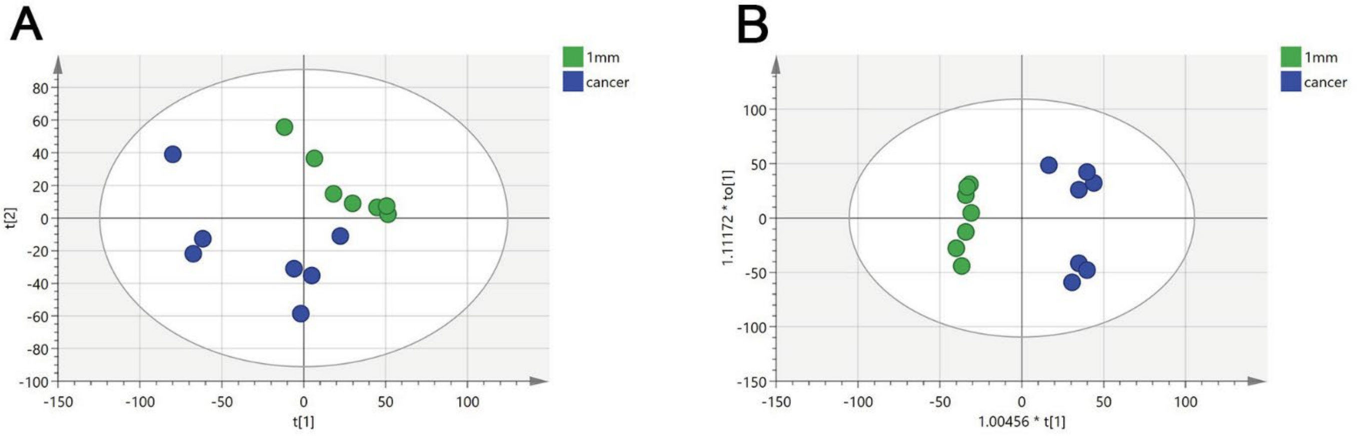

Supplement: Supplementary file 1 [file DataSheet_1.pdf]
